# Microbe-immune interactions: new perspectives on coagulation deficiencies, purpura, and other hemorrhagic conditions under the regulation of the gut microbiota

**DOI:** 10.3389/fimmu.2024.1461221

**Published:** 2024-10-08

**Authors:** Ruhua Ren, Xiaohua Huang, Diu Wei, Qing Guo, Chong Wang, Mengjie Li, Lu Yang, Haiyan Lang, Shana Chen

**Affiliations:** ^1^ The First Clinical Medical College, Beijing University of Chinese Medicine, Beijing, China; ^2^ Department of Hematology, Dongzhimen Hospital, Beijing University of Chinese Medicine, Beijing, China; ^3^ Department of Hematology, International Mongolian Hospital of Inner Mongolia, Hohhot, China

**Keywords:** gut microbiota, immune cells, coagulation defects, Purpura and other hemorrhagic conditions, Mendelian randomization, mediation

## Abstract

**Background:**

The relationship between gut microbiota and coagulation defects, purpura, and other hemorrhagic conditions (CPH) is currently unclear, with causal links yet to be firmly established.

**Objective:**

The causal relationships between gut microbiota and CPH, along with the potential mediating role of immune cells, were studied using Mendelian randomization analysis.

**Methods:**

Data on 412 gut microbiota species, 731 immune cell types, and CPH were methodologically compiled from genome-wide association studies and the FinnGen database. A 2-sample Mendelian randomization approach in 2 stages was used and the causal links between gut microbiota and CPH were statistically analyzed, assessing the potential mediation by immune cells. Sensitivity and reliability were ensured through heterogeneity and pleiotropy tests.

**Results:**

The abundance of *Alistipes putredinis* (odds ratio [OR]=0.77, 95% confidence interval [CI] 0.64–0.93, *P*=0.006) was negatively correlated with CPH, whereas the abundance of *Bacteroides stercoris* (OR=1.25, 95%CI 1.09–1.45, *P*=0.002) was positively correlated with the risk of CPH. There was no evidence of reverse causality or the potential mediating effects of 731 immune cell types. The abundance of Proteobacteria (OR=0.81, 95%CI 0.71–0.92, *P*=0.001) and *Coprococcus* sp. ART55/1 (OR=0.87, 95%CI 0.80–0.96, *P*=0.005) was negatively associated with the risk of CPH, whereas the abundance of Enterobacteriales/Enterobacteriaceae (OR=1.36, 95%CI 1.12–1.64, *P*=0.002) was positively correlated with the risk of CPH, with no evidence of reverse causality. Furthermore, CD38 levels on CD3-CD19 cells can serve as a mediating factor for the influence of Proteobacteria on the pathogenesis of CPH, with a mediating effect ratio of 7.26%.

**Conclusions:**

An increase in Proteobacteria abundance leads to a decrease in CD38 expression on CD3-CD19- cells, thereby reducing the risk of developing CPH. CD3 expression on naive CD4+ in mature T cells serves as a mediating factor for the influence of Enterobacteriales/Enterobacteriaceae on the pathogenesis of CPH, whereas IgD CD38br AC expression on B cells serves as a mediating factor for the influence of *Coprococcus* sp. ART55/1 on the pathogenesis of CPH. The mediating effect is opposite to the overall trend and has a relatively small impact. No significant heterogeneity or pleiotropy was observed.

## 1 Introduction

Coagulation defects, purpura, and other hemorrhagic conditions (CPH) is a category derived from the World Health Organization’s revision of the International Statistical Classification of Diseases and Related Health Problems, 10th Edition (1). It includes disseminated intravascular coagulation, hereditary factor VIII deficiency, hereditary factor IX deficiency, and CPH. CPH is a bleeding disorder caused by immune deficiencies or abnormalities that often manifests as skin and mucosal hemorrhages. Severe cases can potentially lead to severe anemia, heart and kidney failure, sepsis, and shock (1, 2).

The gut microbiota plays a crucial role in maintaining immune system homeostasis (3). Dysbiosis of the gut microbiota can influence the occurrence of CPH by affecting immune homeostasis and tolerance (4). Immune cells act as intermediary factors between the gut microbiota and CPH; however, the specific mechanisms and effects are currently unclear. The associations are easily influenced by confounding factors such as age, environment, dietary habits, and lifestyle, which can limit causal inferences and the analysis of immune cells as mediators.

Mendelian randomization (MR) is a methodological approach based on genetic variation to determine causal inference. It uses the influence of genotype on phenotype to infer the impact of exposure factors on outcomes, circumventing potential confounding factors (5). MR has been extensively used to determine causal relationships between the gut microbiota and conditions such as liver disease, metabolic disorders, autoimmune diseases, and tumors (6, 7).

In this study, 412 types of gut microbiota were selected as exposure factors, 731 immune cells as potential mediators, and CPH as the outcome. A 2-step, 2-sample MR method was used to investigate the causal relationship between gut microbiota abundance and CPH incidence and the potential mediating effects of immune cells.

## 2 Data and methods

### 2.1 Study design

**Figure 1:**
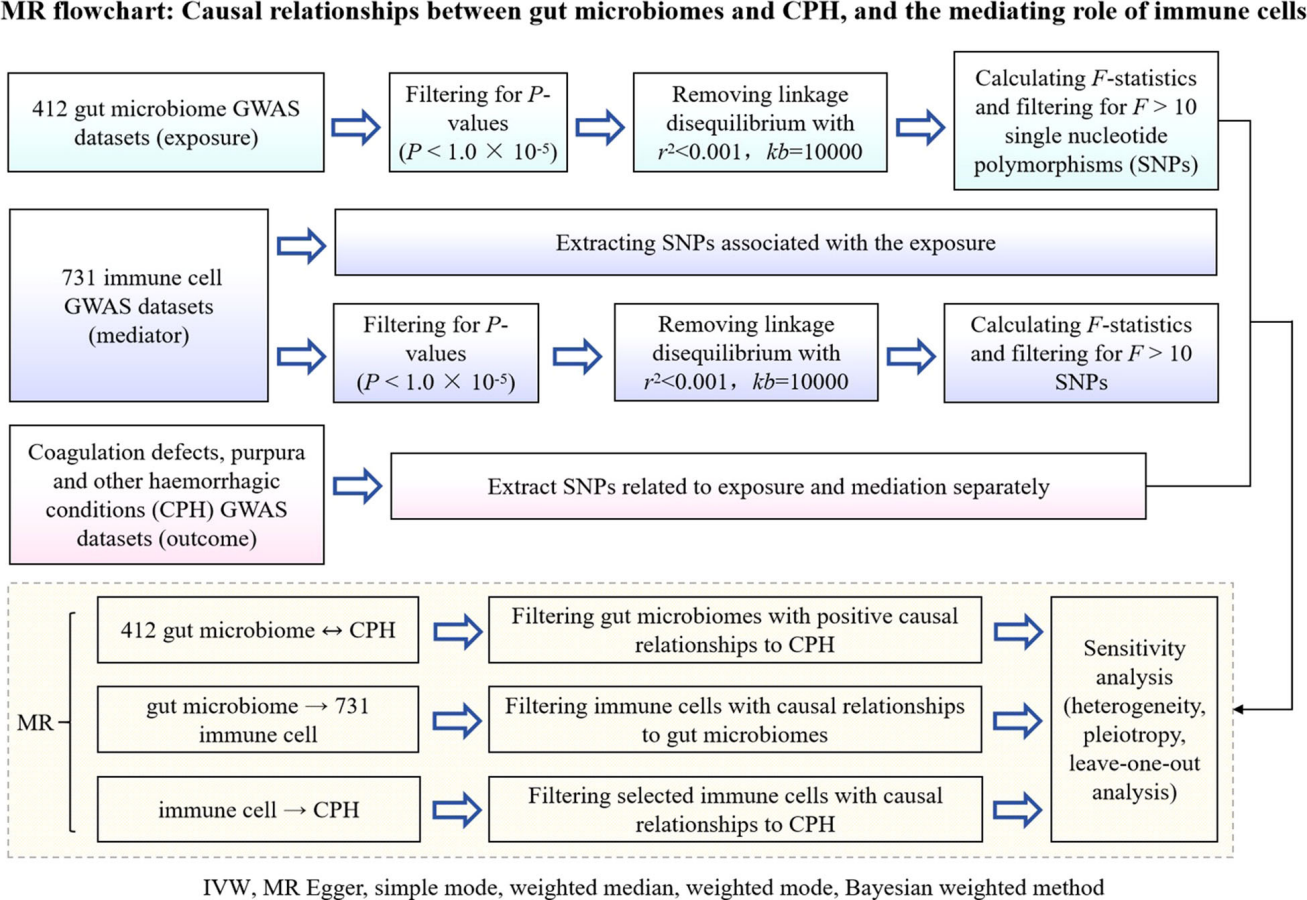
Flowchart of MR analysis for the causal relationship between gut microbiota and CPH, and the mediating role of immune cells. GWAS is genome wide association study; CPH is Coagulation defect, purpura, and other harmonic condition; MR is Mendelian randomization; IVW is Inverse Variance Weighted; SNPs are single nucleotide polymers.

A 2-step, 2-sample Mendelian randomization approach was employed, utilizing gut microbiota as the exposure, immune cells as the mediator, and chronic purpura hemorrhagic as the outcome. Single nucleotide polymorphisms (SNPs) significantly associated with gut microbiota and immune cell levels were selected as instrumental variables to analyze the causal relationship between the abundance of gut microbiota and the incidence of CPH as well as the potential mediating effect of immune cells (**Figure 1** Flowchart).

This mediation analysis required the following 3 conditions to be fulfilled: 1) a causal relationship between the exposure and the mediator; 2) a causal relationship between the mediator and the outcome; and 3) a positive causal relationship between the exposure and the outcome without reverse causality.

The MR analysis was based on the following 3 assumptions (8): 1) the association assumption, where the instrumental variable is strongly related to the predicted exposure; 2) the independence assumption, where the instrumental variable is independent of any confounders related to the exposure, mediator, and outcome; and 3) the exclusivity assumption, where the instrumental variable can only influence the outcome through the exposure or mediator.

### 2.2 Time and location

This study was conducted in the Department of Hematology at Dongzhimen Hospital of Beijing University of Chinese Medicine between March and April 2024.

### 2.3 Data

All gut microbiota and immune cell data were obtained from the summary statistics of genome-wide association studies (GWAS) databases ( *https://www.ebi.ac.uk/gwas/* ). Gut microbiota data, numbered “*GCST90027446-GCST90027857*,” included 412 types of gut microbiota (9), whereas immune cell data, numbered “*GCST90001391-GCST90002121*,” included 731 types of immune cells. CPH data were downloaded from the Finnish database R10 version ( *https://www.finngen.fi/en/access_results* ), numbered “*D3_COAGDEF_PURPUR_HAEMORRHAGIC*,” and included subcategories such as disseminated intravascular coagulation, hereditary factor VIII deficiency, hereditary factor IX deficiency, other coagulation defects, purpura, and other harmonic conditions. The sample size was 412,181, with 6,419 assigned to the disease group and 405,762 to the control group. Detailed data sources are provided in **Table 1**.

**Table 1:** Data sources.

Type	Title	Population	Database	Sample Size	Download Link
Exposure	412 Gut Microbiota IDs	European	GWAS	7738	http://ftp.ebi.ac.uk/pub/databases/gwas/summary_statistics/
Mediator	713 Immune Cell IDs		GWAS	3757	
Outcome	CPH		FinnGen	412181	https://storage.googleapis.com/finngen-public-data-r10/summary_stats/finngen_R10_D3_COAGDEF_PURPUR_HAEMORRHAGIC.gz

### 2.4 Experimental methods

#### 2.4.1 Selection of instrumental variables

Initially, SNPs closely associated with gut microbiota and immune cells (*P*<1.0×10^-5^) were selected as instrumental variables. Subsequently, linkage disequilibrium analysis was conducted based on European genomic sample data with parameters set at *r^2^* <0.001 and *kb*=10,000 to ensure the independence of the selected SNP loci and reduce the impact of pleiotropy. Lastly, the strength of the instrumental variables was assessed by calculating the *F*-statistic, where *F*<10 indicated the presence of weak instrumental variables and *F*>10 indicated the absence of weak instrumental variable bias. Variables with *F*<10 were excluded (10).

The formula for the *F*-statistic is “ $F=\frac{\left(N-k-1\right)\times R^{2}}{k\times \left(1-R^{2}\right)}$ “, where *N* is the sample size of the exposure GWAS data, *K* is the number of SNPs, and *R^2^* is the variance in exposure explained individually by each instrumental variable. The formula for *R^2^* is “ $R^{2}=2\times \left(1-MAF\right)\times MAF\times \frac{\beta^{2}}{se^{2}}$ “, where β is the effect size of the allele, *EAF* is the minor allele frequency, and *se* is the standard deviation (11).

#### 2.4.2 Mendelian randomization analysis

MR analysis was used in this study to investigate the causal relationship between the gut microbiome and CPH. MR uses genetic variations to infer causality, leveraging the influence of genotypes on phenotypes to deduce the impact of exposure factors on outcomes, thereby circumventing common confounding factors. The results were evaluated based on the odds ratio (OR) and 95% confidence intervals (CIs), using heterogeneity tests and horizontal gene pleiotropy tests to determine the stability and reliability of the results (12).

The following methods were selected to ensure the rigor of our analysis (13):

1) Inverse Variance Weighted (IVW) method: This is the most commonly used basic technique for MR analysis. It combines multiple instrumental variables (genetic variants associated with exposure) and estimates the overall effect through inverse variance weighting. The advantage of the IVW method is its ability to enhance the precision of estimates that are particularly evident when multiple independent instrumental variables are available. However, it assumes that all instrumental variables meet the following 3 fundamental assumptions of MR: association, independence, and exclusivity. IVW may yield biased estimates when horizontal pleiotropy exists (where instrumental variables influence outcomes through other pathways).

2) MR Egger Regression: This method is primarily used to detect and correct horizontal pleiotropy, not relying on the assumption that all instrumental variables satisfy the exclusivity condition. By analyzing the propensity of instrumental variables and outcomes using linear regression, MR Egger regression can assess and adjust for bias resulting from horizontal pleiotropy. Egger Regression provides a robust alternative when horizontal pleiotropy is present.

3) Weighted Median Method: This is a more robust method of estimation than IVW that can provide consistent estimates even if half of the instrumental variables are ineffective. The weighted median method combines the effect sizes of instrumental variables and estimates the overall effect by calculating the weighted median, demonstrating better stability in the face of potential horizontal pleiotropy.

4) Weighted Mode Method: Similar to the weighted median method but with a different weighting scheme, the weighted mode method offers additional robustness checks, typically when both IVW and weighted median methods show consistency.

5) Bayesian Weighted MR: This is a Bayesian statistical weighted method that allows the inclusion of prior information during the estimation process to better adjust for model uncertainty. Bayesian weighted MR provides more precise CIs and performs well with small sample sizes (14).

These methods were selected because they offer complementary advantages under different circumstances. The IVW method provides the most efficient estimates in the absence of horizontal pleiotropy, whereas the MR Egger regression and the weighted Median method are excellent in handling horizontal pleiotropy and ensure estimation robustness, respectively. By integrating these methods, we could achieve a comprehensive and robust evaluation of the results, enhancing the reliability and validity of the study. Bayesian weighted MR, as a supplementary analysis, further improves the robustness of the results, especially when the small sample size is small. Therefore, the choice of these methods ensures that our research outcomes are not only highly statistically powerful but also resistant to common analytical pitfalls, such as horizontal pleiotropy and potential reverse causality.

### 2.5 Main observational indicators

MR methods were used in this study to analyze the following 3 aspects: 1) the causal relationship between the abundance of gut microbiota and the incidence of CPH; 2) whether immune cell levels can serve as a mediating factor between the abundance of gut microbiota and the incidence of CPH; and 3) to what extent immune cells mediate the influence of the abundance of gut microbiota on the incidence of CPH.

### 2.6 Statistical analysis

**Figure 2:**
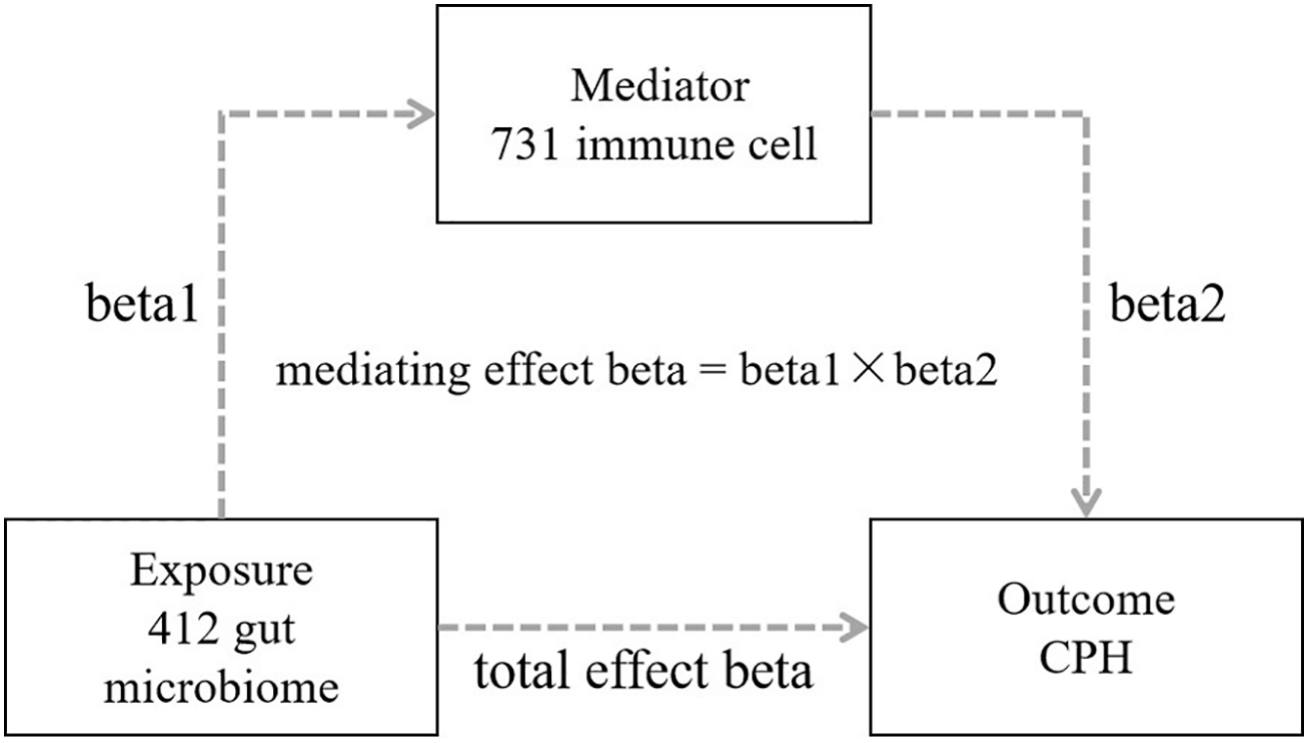
Schematic diagram of mediational MR analysis.

*R* software (version 4.1.3), primarily utilizing packages such as “*TwoSampleMR*”, “*ggplot2*”, and “*forestploter*” was used for statistical analysis and plotting. The process included the following 4 steps: 1) Conducting a 2-step, 2-sample MR bidirectional batch analysis between 412 types of gut microbiota and CPH to screen for gut microbiota with statistically significant results in the forward analysis (*P*<0.05) and for no statistical significance in the reverse analysis (*P*>0.05), while calculating the total effect beta value; 2) Conducting a 2-step, 2-sample MR analysis between the screened gut microbiota and 731 types of immune cells to identify immune cells associated with the gut microbiota, and obtaining the effect value beta1; 3) Conducting batch MR analysis between the identified immune cells associated with the gut microbiota and CPH, screening for immune cells related to CPH, determining the mediating factor, and obtaining the effect value beta2; and 4) Calculating the mediating effect and direct effect: mediating effect beta = beta1 × beta2; direct effect beta = total effect beta–mediating effect beta (**Figure 2**).

## 3 Results

### 3.1 Causal relationship and sensitivity analysis between gut microbiota and CPH

**Figure 3:**
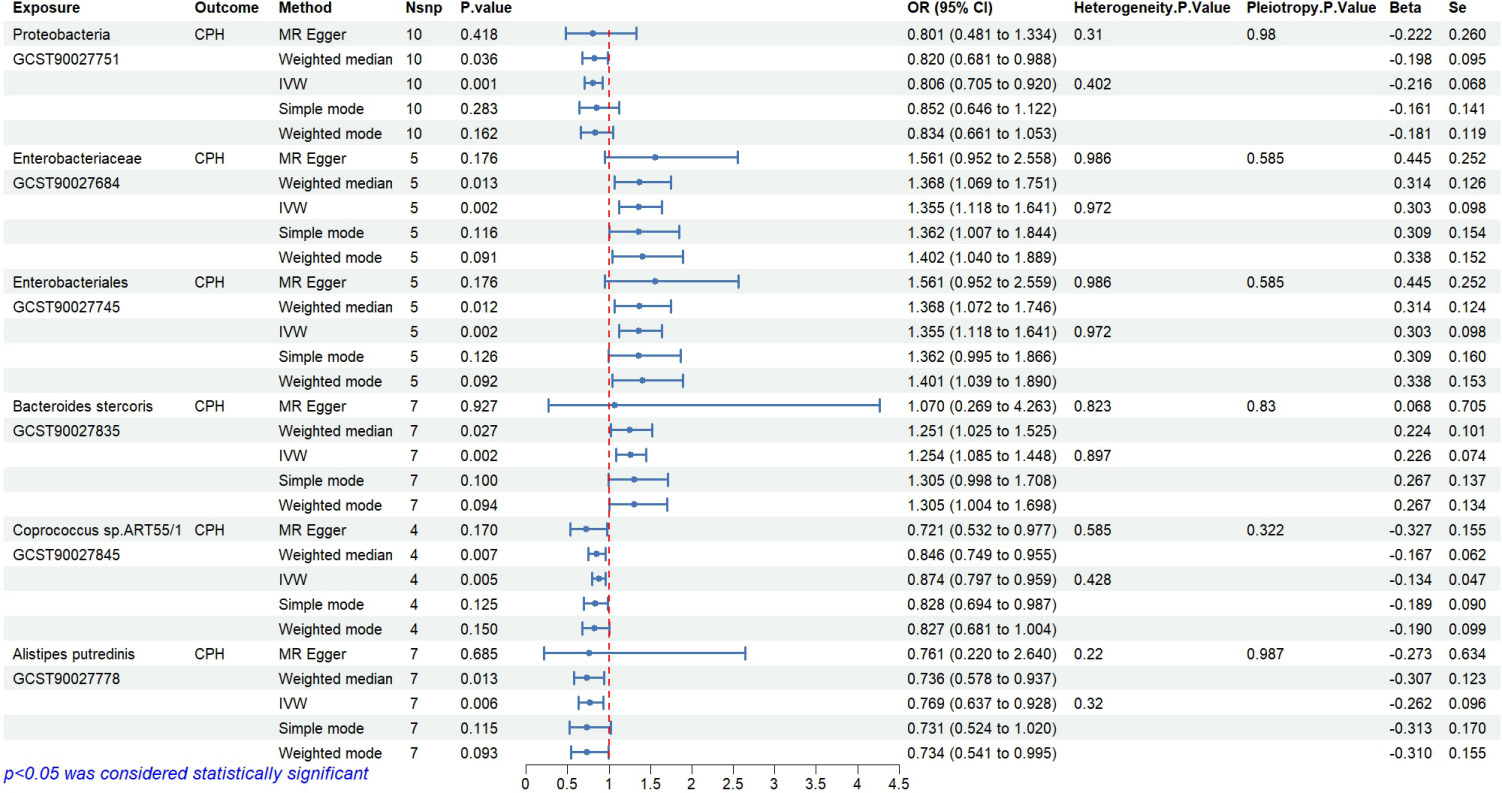
Causal relationships and sensitivity analysis between gut microbiota and CPH.

**Figure 4:**
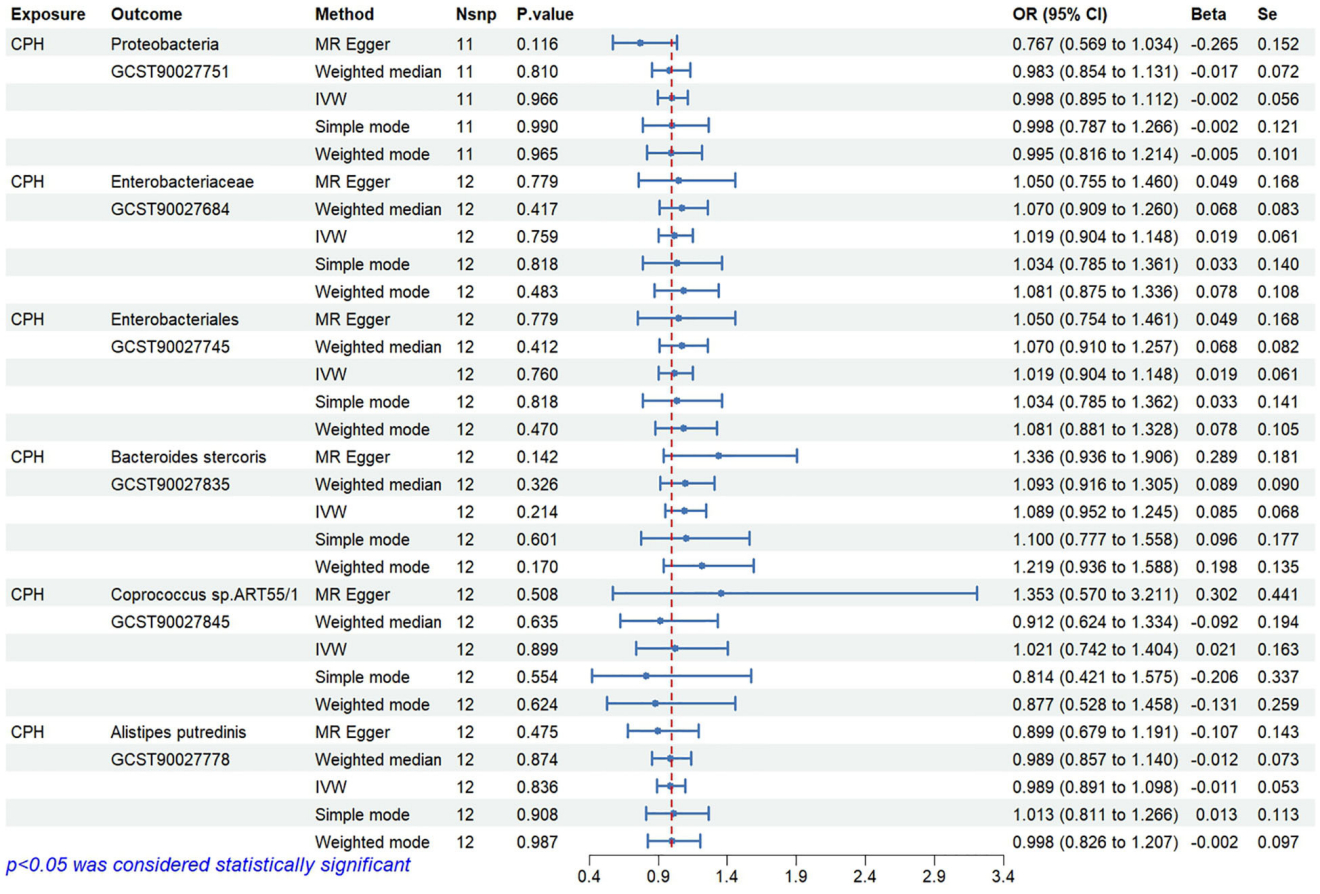
Reverse causal relationships between gut microbiota and CPH.

The causal relationships between 412 types of gut microbiota and CPH were examined using the 2-step, 2-sample MR analysis. Nineteen types of gut microbiota associated with the disease (*P*<0.05, see Appendix 1 for details) were identified and further narrowed down to 6 significant types (*P*<0.01; GWAS IDs and corresponding scientific names listed in **Table 2**). Heterogeneity and pleiotropy tests showed no significant heterogeneity among the instrumental variables (*P*>0.05, **Figure 3**). Reverse MR analysis indicated no causal effect of CPH on these 6 types of gut microbiota (**Figure 4**). The MR effect forest plots, SNP scatter plots, and leave-one-out analysis plots are provided in **Appendix 2**.

**Table 2:** GWAS ID information and scientific names of 6 significant gut microbiota (*P*<0.01).

Gut Microbiota ID	corresponding scientific name
GCST90027751	k_Bacteria.p_Proteobacteria
GCST90027684	k_Bacteria.p_Proteobacteria.c_Gammaproteobacteria.o_Enterobacteriales.f_Enterobacteriaceae
GCST90027745	k_Bacteria.p_Proteobacteria.c_Gammaproteobacteria.o_Enterobacteriales
GCST90027835	Bacteria.p_Bacteroidetes.c_Bacteroidia.o_Bacteroidales.f_Bacteroidaceae.g_Bacteroides.s_Bacteroides_stercoris
GCST90027845	k_Bacteria.p_Firmicutes.c_Clostridia.o_Clostridiales.f_Lachnospiraceae.g_Coprococcus.s_Coprococcus_sp_ART55_1
GCST90027778	k_Bacteria.p_Bacteroidetes.c_Bacteroidia.o_Bacteroidales.f_Rikenellaceae.g_Alistipes.s_Alistipes_putredinis

#### 3.1.1 Abundance of Proteobacteria, *Coprococcus* sp.ART55/1 and *Alistipes putredinis* is negatively associated with CPH risk

The results indicate that the abundance of Proteobacteria (OR=0.81, 95%CI 0.71–0.92, *P*=0.001), *Coprococcus* sp.ART55/1 (OR=0.87, 95%CI 0.80–0.96, *P*=0.005), and *Alistipes putredinis* (OR=0.77, 95%CI 0.64–0.93, *P*=0.006) is negatively associated with the risk of CPH.

#### 3.1.2 Abundance of Enterobacteriaceae/Enterobacteriales and *Bacteroides stercoris* is positively associated with CPH risk

The results show that the abundance of Enterobacteriaceae/Enterobacteriales (OR=1.36, 95%CI 1.12–1.64, *P*=0.002) and *Bacteroides stercoris* (OR=1.25, 95%CI 1.09–1.45, *P*=0.002) is positively associated with the risk of CPH.

### 3.2 Bayesian validation confirms the causal relationship between specific gut microbiota and CPH

**Figure 5:**
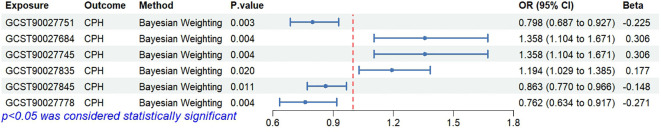
Causal relationships between gut microbiota and CPH based on Bayesian weighting method.

Bayesian weighting was used to validate the results from **Figure 3** (see **Figure 5**). Statistical analysis revealed significant *P*-values for Proteobacteria (GCST90027751, *P*=0.003), Enterobacteriales/Enterobacteriaceae (GCST90027684/GCST90027745, *P*=0.004), *Bacteroides stercoris* (GCST90027835, *P*=0.020), *Coprococcus* sp. ART55/1 (GCST90027845, *P*=0.011), and *Alistipes putredinis* (GCST90027778, *P*=0.004). All *P*-values were <0.05, confirming the causal relationship between these 6 types of gut microbiota and CPH and supporting the reliability of the results in **Figure 1**.

### 3.3 Causal relationship between gut microbiota and immune cells

Subsequent MR analysis identified immune cells causally related to the 6 types of gut microbiota (**Appendix 3**), including 26 immune cells related to Proteobacteria (ID GCST90027751, *P*<0.05); 63 immune cells related to Enterobacteriales/Enterobacteriaceae (IDs GCST90027684 and GCST90027745, *P*<0.05); 25 immune cells related to *B. stercoris* (ID GCST90027835, *P*<0.05); 27 immune cells related to *Coprococcus* sp. ART55/1 (ID GCST90027845, *P*<0.05); and 25 immune cells related to *A. putredinis* (ID GCST90027778, *P*<0.05). The MR effect forest plots, SNP scatter plots, and leave-one-out analysis plots are provided in **Appendix 2**.

### 3.4 Levels of CD38 on CD3- CD19- cell, CD3 on naive CD4+, CD40 on CD14+ CD16- monocyte and IgD- CD38br AC correlate positively with CPH risk

**Figure 6:**
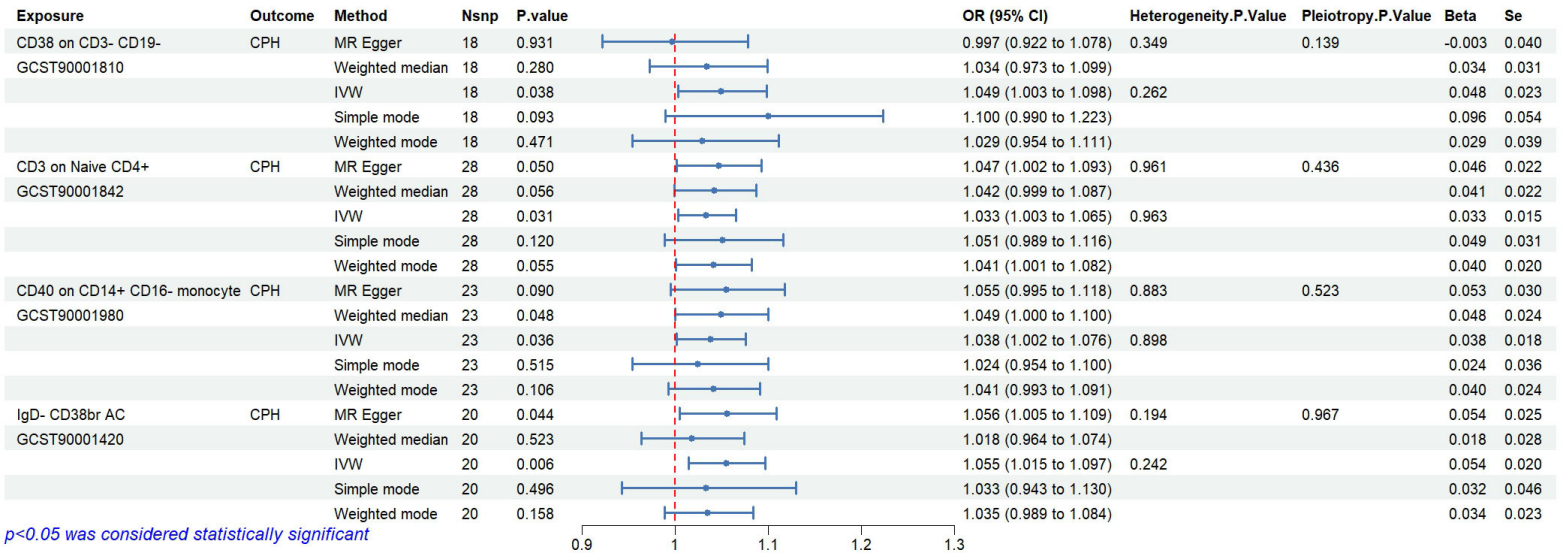
Causal relationships and sensitivity analysis between immune cells and CPH.

Statistical analysis of the 6 groups of immune cells identified in Section 2.3 using CPH data revealed 4 immune cells causally related to CPH. The results are as follows (**Figure 6**): CD38 expression on CD3-CD19- cells (ID GCST90001810) related to Proteobacteria (ID GCST90027751, *P*<0.05); CD3 expression on naive CD4+ cells (ID GCST90001842), and CD40 expression on CD14+ CD16- cells (ID GCST90001980) related to Enterobacteriales/Enterobacteriaceae (ID GCST90027684/GCST90027745, *P*<0.05); and IgD- CD38br AC in B cells (ID GCST90001420) related to *Coprococcus* sp. ART55/1 (ID GCST90027845, *P*<0.05). Heterogeneity and pleiotropy tests showed no significant heterogeneity among the instrumental variables (*P*>0.05). The MR effect forest plots, SNP scatter plots, and leave-one-out analysis plots are provided in **Appendix 2**.

### 3.5 Bayesian validation confirms the causal relationship between partial immune cells and CPH

**Figure 7:**
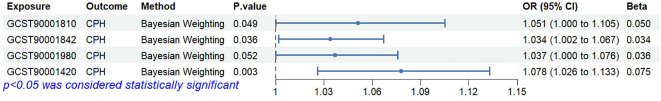
Causal relationships between immune cells and CPH based on Bayesian weighting method.

Bayesian weighting was used to validate the causal relationship between immune cells and CPH as shown in **Figure 6** (see **Figure 7**). Significant *P*-values were obtained for IgD- CD38br AC (GCST90001420, *P*=0.003), CD38 on CD3- CD19- (GCST90001810, *P*=0.049), and CD3 on naive CD4+ (GCST90001842, *P*=0.036). All *P*-values were <0.05, supporting the reliability of these findings. However, the *P*-value for CD40 on CD14+ CD16- monocytes (GCST90001980) was 0.052, which exceeded 0.05. Thus, this result was excluded.

### 3.6 Mediation role of immune cells in MR analysis

Mediation effect calculations in MR analysis are presented in **Table 3**. Our findings indicated that the mediation effect proportion be calculated as 7.26% only when the exposure factor is Proteobacteria (GCST90027751), the mediator is the expression of CD38 on CD3- CD19- (GCST90001810), and the outcome is CPH.

**Table 3:** MR analysis of the mediation role of immune cells in the relationship between gut microbiota and coagulation defects, purpura, and other hemorrhagic conditions.

Exposure	Mediator	Outcome	Total Effect Beta	Mediation Beta	Proportion of Mediation Effect
Gut Microbiota ID	Immune Cell ID				
GCST90027751	GCST90001810	CPH	-0.216	-0.016	7.26%
GCST90027684	GCST90001842	CPH	0.303	-0.009	/
GCST90027745	GCST90001842	CPH	0.303	-0.009	/
GCST90027845	GCST90001420	CPH	-0.134	0.008	/

## 4 Discussion

CPH is a collective term for harmonic conditions caused by deficiencies or abnormalities in hemostasis. Patients often exhibit varying degrees of skin, mucosal, joint, or visceral bleeding symptoms. Rapid clinical assessment and timely treatment are crucial in improving patient outcomes (1). Dysbiosis of the gut microbiota can increase the risk of CPH (15). Providing nutritional support to the gut microbiota in patients with CPH can generate a defensive barrier in the intestinal mucosa, effectively enhance immune function, increase platelet levels, prevent bleeding, and improve post–treatment outcomes (16). The immune system plays a crucial role in this process; however, the causal relationship and specific mechanisms are unclear.

The gut microbiota in healthy individuals is primarily composed of the phyla Bacteroidetes, Firmicutes, Proteobacteria, and Actinobacteria, with Bacteroidetes and Firmicutes being dominant and accounting for 90% of the human gut microbiota (17). The gut microbiota can act as pathogenic bacteria causing infections or as commensal bacteria maintaining intestinal homeostasis. They can also interact directly or indirectly with immune cells through metabolic products and cell surface molecules, influencing the intensity and direction of immune responses (18, 19).

### 4.1 Causal relationship between gut microbiota and CPH

The overgrowth of Proteobacteria and Bacteroidetes in the gut suggests an imbalance in the microbial community structure (20). Compared with healthy individuals, patients with immune thrombocytopenia in CPH exhibit increased relative abundances of Proteobacteria and Bacteroidetes, and decreased abundances of Firmicutes and Actinobacteria (21). Consequently, it has been proposed that an increase in the abundance of Proteobacteria and Bacteroidetes and a decrease in that of Firmicutes can serve as a marker of gut dysbiosis and as a potential diagnostic criteria for metabolic, immune, and inflammatory diseases (22). The results of this meta-analysis showed that the abundance of *B. stercoris* (OR=1.25, 95%CI 1.09–1.45, *P*=0.002) was positively correlated with the risk of CPH, whereas the abundance of Proteobacteria (OR=0.81, 95%CI 0.71–0.92, *P*=0.001) was negatively correlated with the risk of CPH. The abundance of Enterobacteriaceae/Enterobacteriales (OR=1.36, 95%CI 1.12–1.64, *P*=0.002) was positively correlated with the risk of CPH and there was no reverse causality. An increase in the abundance of Proteobacteria may be a protective factor against developing CPH, contrary to that reported previously.

### 4.2 The mediating role of immune cells

**Figure 8:**
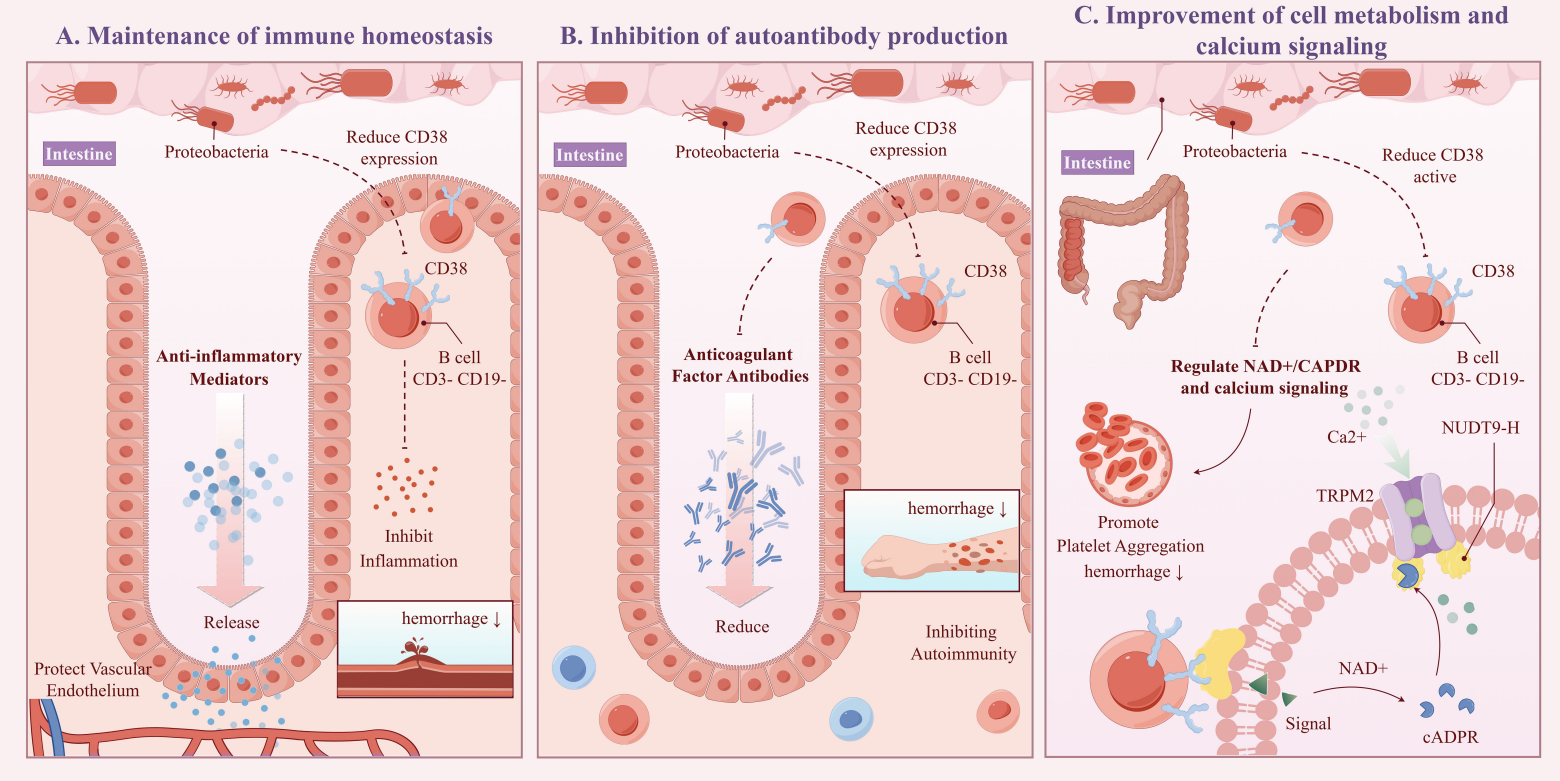
Schematic diagram of the potential mechanism for CPH under the influence of proteobacteria (ID:YUSSI95293). Including: Maintenance of immune homeostasis **(A)**, Inhibition of autoantibody production **(B)**, Improvement of cell metabolism and calcium signaling **(C)**.

The “CD3-CD19-” cell phenotype typically does not correspond to typical mature B cells, potentially representing early B cell precursors, atypical B cells, or certain B cell subsets in specific activation states (23). CD38 is a multifunctional glycoprotein highly expressed in various immune cells. In B cells, CD38 interacts with the B cell receptor signaling pathway, promoting B cell activation, proliferation, and differentiation into plasma cells, which produce antibodies (24). CD38 can also influence intracellular NAD+/cADPR levels and calcium signaling through its enzymatic activity (e.g., NADase/ADP-ribosyl cyclase), affecting immune cell function (25). Our study shows that CD38 expression on B cells in the CD3-CD19- phenotype can act as a mediator for Proteobacteria affecting the onset of CPH, with a mediating effect accounting for 7.26%. This effect was manifested as an increase in the abundance of Proteobacteria, leading to a decrease in CD38 expression on CD3-CD19- cells, thereby reducing the risk of CPH. The possible mechanisms include (see **Figure 8**, ID: YUSSI95293): 1) Maintenance of immune homeostasis: An increase in Proteobacteria abundance may downregulate CD38 expression on CD3-CD19- cells by secreting anti-inflammatory mediators, promoting immune tolerance, or direct interaction, thereby inhibiting excessive inflammatory responses and autoimmune attacks, protecting vascular endothelial integrity, and preventing bleeding tendencies caused by inflammation (26); 2) Inhibition of autoantibody production: If CD38 on CD3-CD19- cells are involved in autoantibody production (e.g., anticoagulant factor antibodies), its downregulation may reduce the production of these antibodies, thereby lowering the risk of coagulation disorders due to autoimmune mechanisms (27); 3) Improvement of cell metabolism and calcium signaling: Proteobacteria may influence enzymatic activity in CD38 and its effects on NAD+/cADPR and calcium signaling through modulation of the host metabolic environment or other indirect pathways. A decrease in CD38 expression may help restore intracellular homeostasis, improve platelet and endothelial cell function, and reduce the risk of bleeding (28).

The study results also indicated that CD3 expression on naive CD4+ cells in mature T cells could serve as an intermediary factor for the impact of Enterobacteriales/Enterobacteriaceae in CPH, albeit with an opposite effect on the overall result. Specifically, there was a negative correlation between the abundance of Enterobacteriales/Enterobacteriaceae and CD3 expression on naive CD4+ cells in mature T cells, indicating that an increase in Enterobacteriales/Enterobacteriaceae abundance led to a decrease in CD3 expression in naive CD4+ cells in mature T cells. CD3 expression in naive CD4+ cells in mature T cells was positively correlated with the risk of CPH, indicating that a decrease in CD3 expression in naive CD4+ cells in mature T cells reduced the risk of CPH. Based on this finding, it was hypothesized that an increase in the abundance of Enterobacteriales/Enterobacteriaceae would lower the risk of CPH. However, MR analysis revealed a positive correlation between the abundance of Enterobacteriales/Enterobacteriaceae and the risk of CPH. Therefore, it is believed that the increase in the abundance of Enterobacteriales/Enterobacteriaceae leading to an increased risk of CPH involves multiple mechanisms and that CD3 expression in naive CD4+ cells in mature T cells, as an intermediary factor for the impact of Enterobacteriales/Enterobacteriaceae on CPH, has an opposite and minor effect compared with the overall trend. Similarly, IgD- CD38br AC expression in B cells serves as an intermediary factor for the impact of *Coprococcus* sp. ART55/1 on CPH, with an opposite and minor effect compared with the overall trend.

### 4.3 Future directions

When discussing the potential implications of our research findings in clinical practice, we recognize that the role of the microbiome and immune regulation identified in our study in CPH can provide new perspectives for disease prevention and treatment strategies. These include precision nutrition interventions that tailor dietary plans and prebiotic supplementation to promote the abundance of beneficial gut bacteria, thereby modulating the immune system and reducing bleeding risks, which is a significant improvement over the current generic dietary recommendations. Microbial therapies such as fecal microbiota transplantation (FMT) and probiotic supplementation offer a means to restore the microecological balance of the gut, enhancing immune regulation and reducing bleeding events beyond what standard treatments provide. Developing immunomodulatory drugs that target CD38 represents another step forward and offers adjunctive treatments to control inflammation and prevent vascular damage, addressing an aspect often overlooked in current therapeutic strategies. Specifically, the following points highlight the clinical translation potential of our findings:

1) Precision Nutrition Intervention: Given the association of specific gut microbes such as *A. putredinis*, Proteobacteria, and *Coprococcus* sp. ART55/1 with reduced CPH risk, personalized dietary and prebiotic supplementation plans can be designed to promote the growth of these bacteria. For example, foods rich in dietary fiber can increase the abundance of Proteobacteria, which, in turn, can reduce bleeding risk by modulating CD38 expression in CD3-CD19- cells.

2) Microbiome Therapy: FMT or specific probiotic supplementation aims to reconstruct a healthy gut microbial environment, particularly in patients with CPH who have a microbial imbalance in the gut due to antibiotic use or other reasons (29). By restoring the normal levels of Proteobacteria and *Coprococcus* sp. ART55/1, immune regulation can be indirectly improved and bleeding episodes can be reduced.

3) Immunomodulatory Drugs: Based on the influence of Proteobacteria on CD38 expression, drugs targeting CD38 can be developed to modulate T and B cell functions, inhibit excessive immune responses, and prevent vascular damage and bleeding tendencies (30). For instance, CD38 inhibitors can be used as adjunctive therapies to control inflammation in patients with CPH.

4) Biomarkers and Early Diagnosis: The microbiome characteristics revealed in our study can serve as biomarkers for the early identification and monitoring of CPH. For example, abnormal levels of *B. stercoris*, Enterobacteriales/Enterobacteriaceae, and *Coprococcus* sp. ART55/1 may help predict the development of CPH, allowing for timely intervention.

5) Personalized Treatment Strategies: Personalized treatment plans including medication selection and dosage adjustments can be developed by analyzing the composition of the patient’s gut microbiome (31). For instance, patients with a low abundance of Proteobacteria may require more aggressive immunosuppressant therapy to compensate for their diminished natural immune-regulation capabilities.

In summary, our findings provide novel insights into the prevention and treatment of CPH, emphasizing the importance of the interplay between the microbiome and the immune system. Future research should determine the application of these mechanisms across different populations and validate the efficacy and safety of microbiome interventions in clinical practice. It may be possible to achieve more precise methods of disease management and patient care by integrating microbiome analysis into routine medical practice.

### 4.4 Limitations

This study used a 2-step, 2-sample MR approach to determine the causal associations between 412 gut microbiota species and CPH and elucidate the potential mediating effects of 731 immune cell types. However, our study has the following limitations that warrant consideration:

1) Validity of Genetic Instruments: The reliance on genetic variants as proxies for gut microbiota and immune cell types may introduce bias, as these variants can influence CPH through alternative pathways that are not related to the proposed microbiota or immune cells. To mitigate this, future studies should consider using multiple genetic instruments and performing sensitivity analyses to ensure robustness.

2) Population Specificity: Data sources including GWAS and the FinnGen database are predominantly derived from European populations, potentially limiting the applicability of our findings to non-European demographics. Therefore, future studies that include diverse global populations are essential to expand the investigation and enhance the generalizability of our findings.

3) Temporal Relationship: Our study did not account for temporal changes in the gut microbiota or immune cell composition, which could affect the interpretation of the results.

4) Limited Mediation Analysis: While immune cell mediation was a focus of this study, future studies should also explore other potential mediators such as metabolic factors or cytokine profiles to comprehend the mechanisms linking gut microbiota to CPH.

5) Confounding Factors and Errors: Despite the use of MR, the possibilities of unmeasured confounding factors or statistical errors remain, which could have influenced outcomes.

## 5 Summary

Several significant findings were made in this study elucidating the causal relationship between the gut microbiota and CPH. The abundance of *A. putredinis*, Proteobacteria, and *Coprococcus* sp. ART55/1 was associated with a reduced risk of CPH, whereas the abundance of Enterobacteriales/Enterobacteriaceae and *B. stercoris* was linked to an increased risk of CPH. These results suggested that modulating the balance of specific gut microbes may serve as an effective approach to the prevention and treatment of CPH.

**Figure 9:**
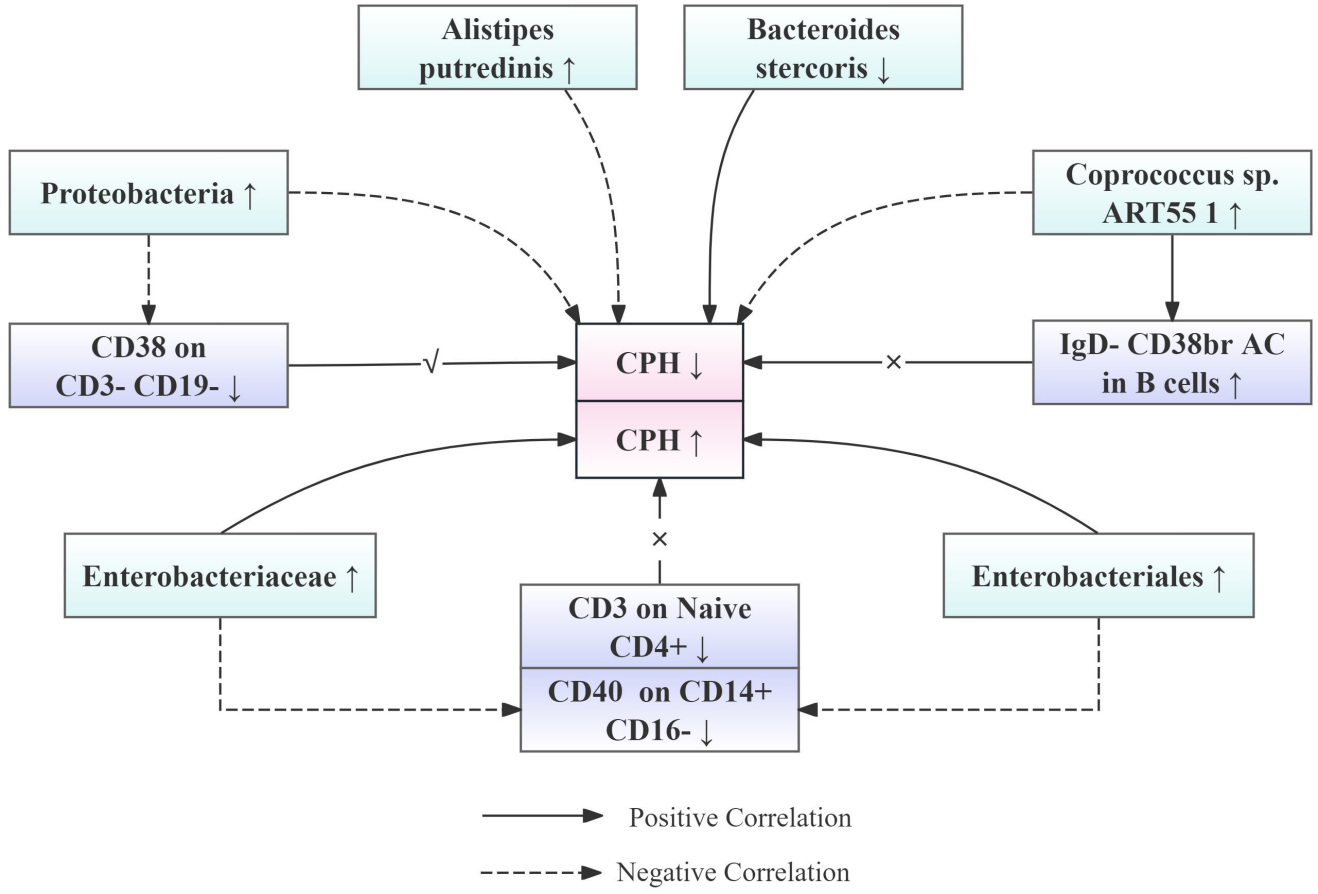
Simplified schematic diagram of the relationship between gut microbiota abundance, immune cell levels, and the risk of CPH onset.

Particularly noteworthy is our discovery of interactions between the microbiota and the immune system, where the impact of the microbiota on CPH may be mediated through the regulation of immune cells. Specifically, CD38 expression in CD3-CD19 cells acts as a mediator in the effect of Proteobacteria in the pathogenesis of CPH, with a mediated proportion of 7.26%. Additionally, CD3 expression in Naive CD4+ mature T cells and IgD- CD38br AC expression in B cells serve as mediators for the effects of Enterobacteriales/Enterobacteriaceae and *Coprococcus* sp. ART55/1 abundance on CPH, respectively, albeit with opposite trends and smaller effects. To facilitate the understanding of the relationships among our proposed results, the team has created a simplified schematic diagram (See **Figure 9**).

Based on these findings, several future research directions can be proposed that aim at translating our results into clinical practice and providing new strategies for the prevention and treatment of CPH. First, personalized nutritional interventions can become feasible through customized dietary plans and prebiotic supplementation wherein the abundance of *A. putredinis*, Proteobacteria, and *Coprococcus* sp. ART55/1 can be increased to modulate the immune system and reduce bleeding tendencies. Second, microbial therapies such as FMT and specific probiotic supplementation can help restore the microecological balance in the gut of patients with CPH, indirectly improving immune regulation and reducing bleeding events. Third, developing immunomodulatory drugs targeting CD38 can serve as adjunctive treatments to control inflammation in patients with CPH, preventing vascular damage and bleeding tendencies. Lastly, microbial signatures can be used as biomarkers for early diagnosis, with abnormal levels of Enterobacteriales/Enterobacteriaceae and *B. stercoris* potentially indicating the development of CPH and enabling timely intervention.

## Data Availability

The original contributions presented in the study are included in the article/**Supplementary Material**. Further inquiries can be directed to the corresponding authors.
