# Supplementary material for: Microbe-immune interactions: new perspectives on coagulation deficiencies, purpura, and other hemorrhagic conditions under the regulation of the gut microbiota
Source: Front Immunol. 2024 Oct 8;15:1461221. doi: 10.3389/fimmu.2024.1461221 (PMC11493621; doi:10.3389/fimmu.2024.1461221)
Supplement: Supplementary file 2 [file Table2.docx]

**Microbe-Immune Interactions: New Perspectives on Coagulation Deficiencies, Purpura, and Other Hemorrhagic Conditions Under the Regulation of the Gut Microbiota**

**Appendix 2**

Due to space constraints, all MR effect forest plots, SNP scatter plots, and leave-one-out analysis plots mentioned in the article will be displayed in Appendix 2.

**1 MR effect forest plots**


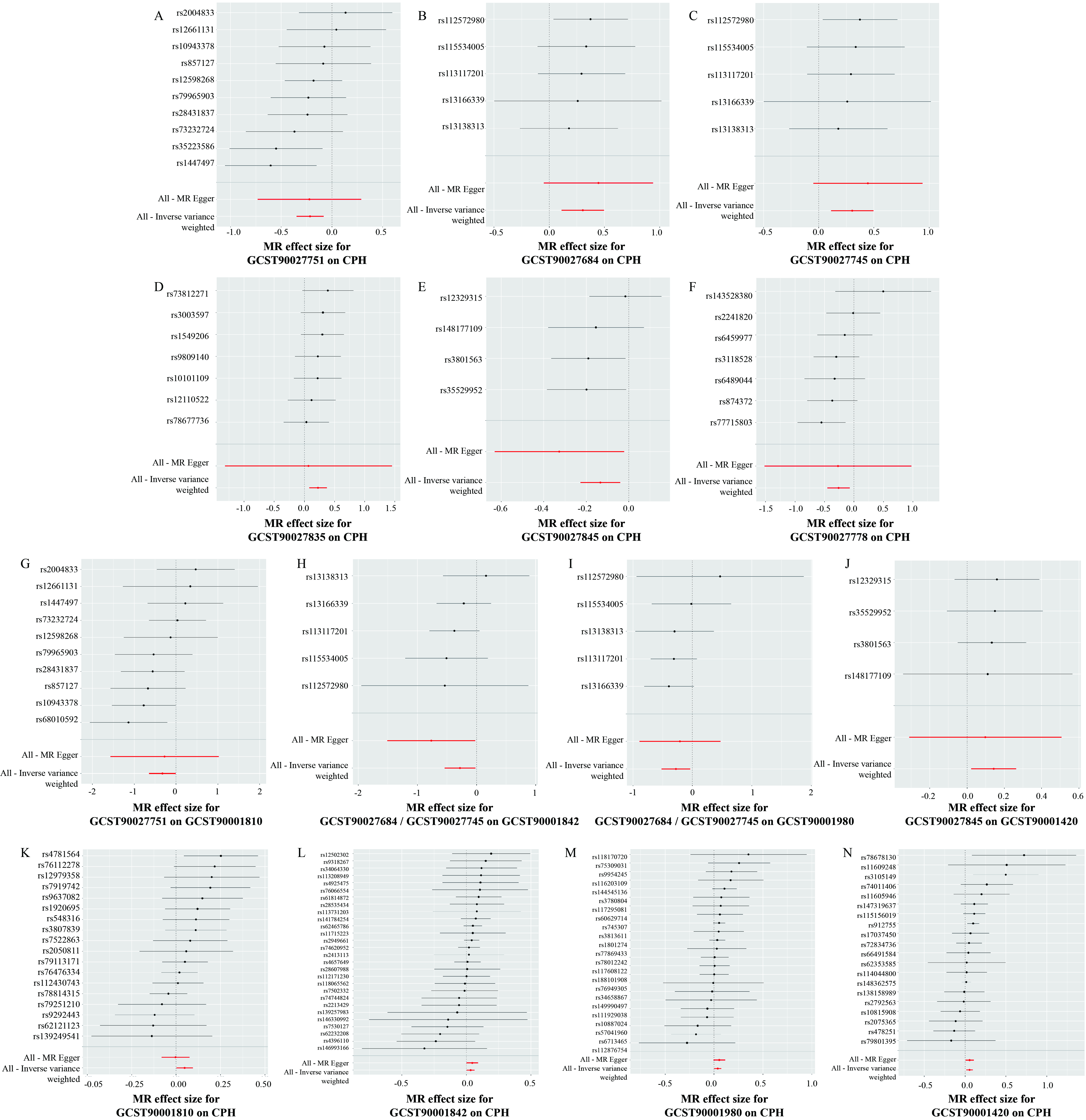


Figure 1 Forest plot of MR effects between gut microbiota, immune cells, and CPH.

Note: CPH refers to Coagulation defects, purpura and other hemorrhagic conditions.

**2 SNP scatter plots**


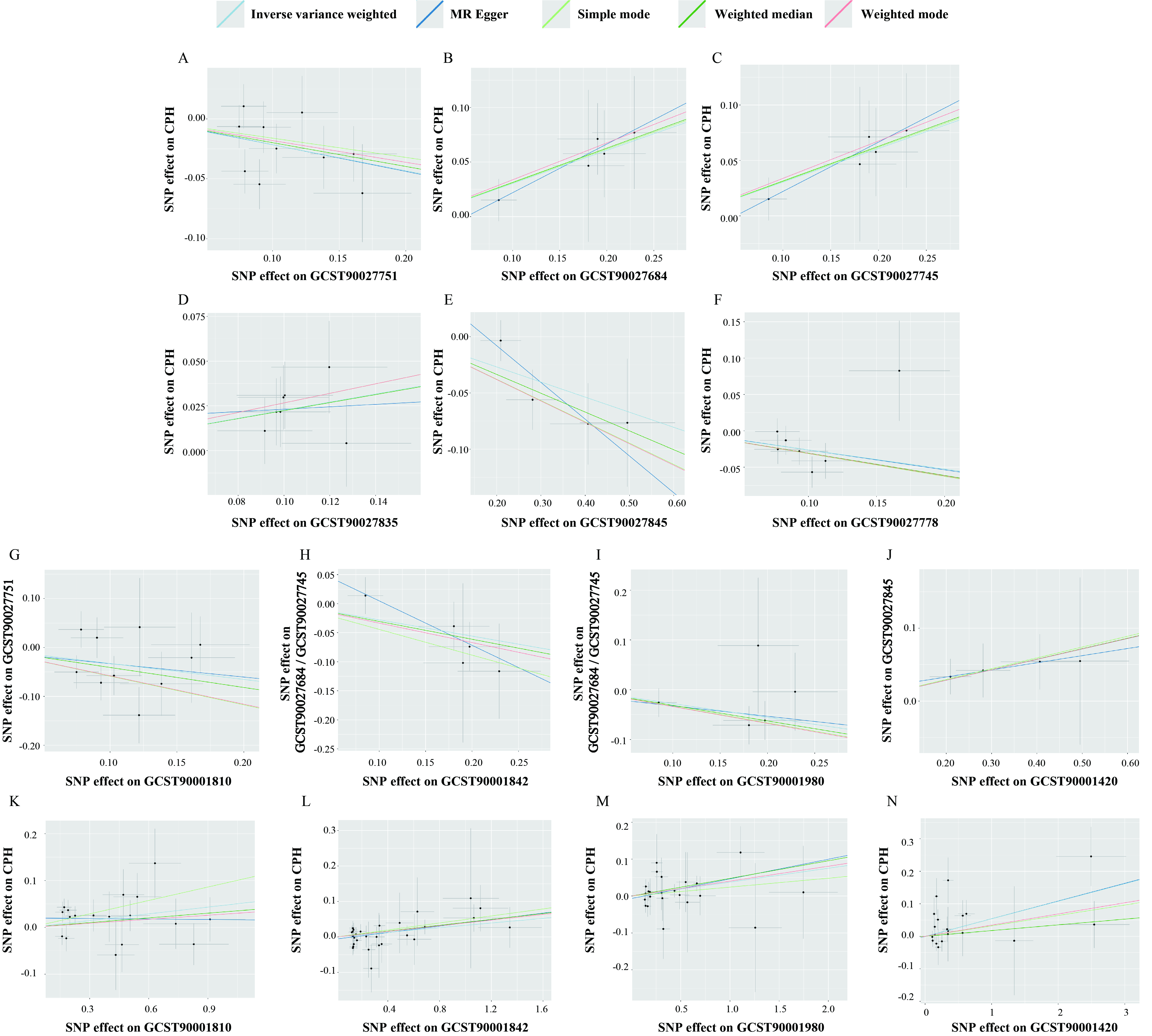


Figure 2 SNP scatter plots between gut microbiota, immune cells, and CPH.

Note: CPH refers to Coagulation defects, purpura and other hemorrhagic conditions.

**3 Leave-one-out analysis plots**


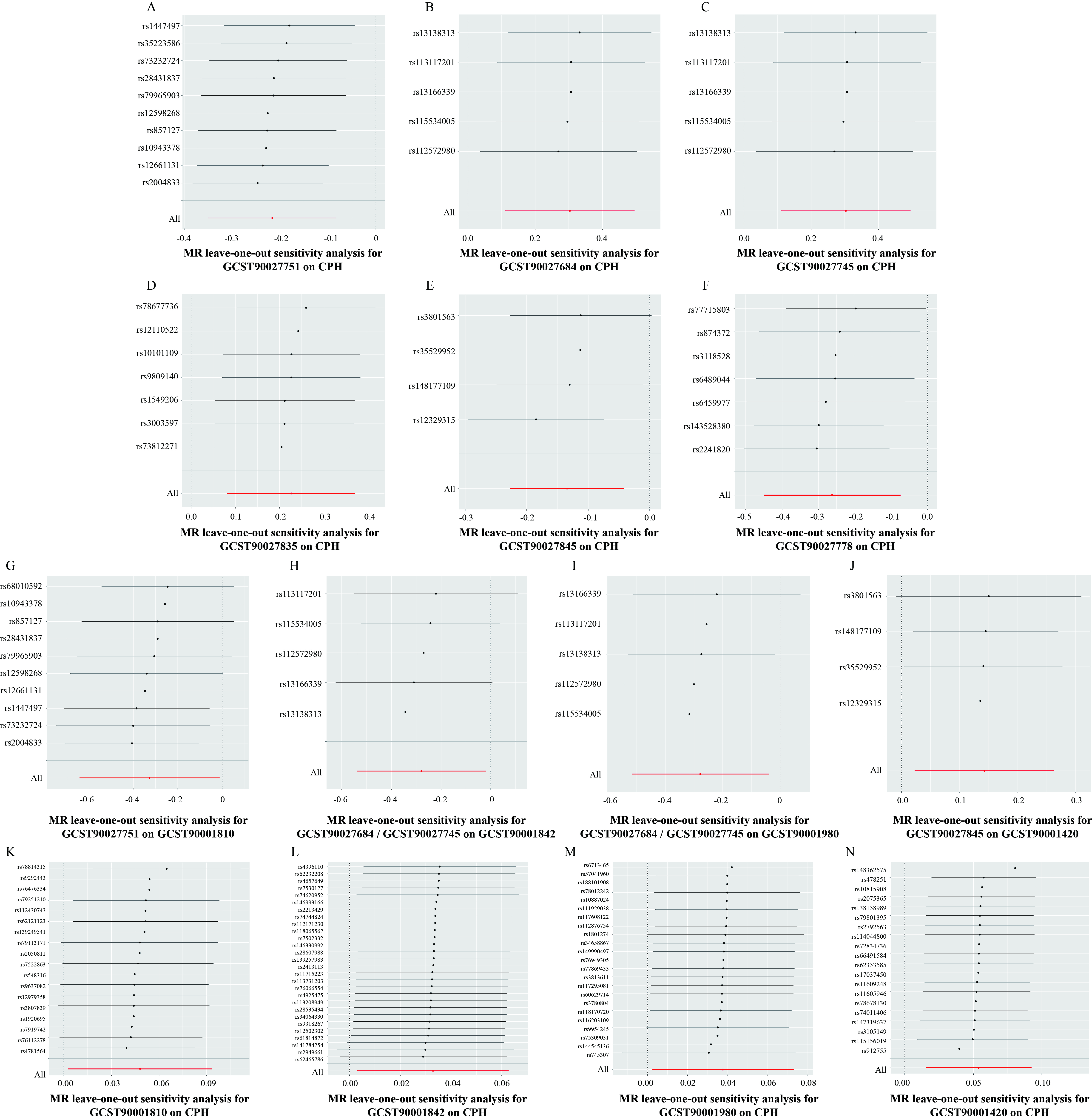


Figure 3 Leave-one-out analysis plots between gut microbiota, immune cells, and CPH.

Note: CPH refers to Coagulation defects, purpura and other hemorrhagic conditions.
